# Effect of Premarital Education on the Quality of Life of Female Partners: A Cross-Sectional Study

**DOI:** 10.7759/cureus.32186

**Published:** 2022-12-04

**Authors:** Nedaa M Bahkali, Ghaida A Eissa, Farah M Alharbi, Fatmah A Alzahrani, Fawaz E Edris, Nahla K Ibrahim

**Affiliations:** 1 Obstetrics and Gynecology, King Abdulaziz University, Jeddah, SAU; 2 Medical School, King Abdulaziz University, Faculty of Medicine, Jeddah, SAU; 3 Medical School, King Abdulaziz University Hospital, Jeddah, SAU; 4 Obstetrics and Gynecology, Umm Al Qura University, Makkah, SAU; 5 Epidemiology, King Abdulaziz University, Jeddah, SAU

**Keywords:** saudi, partner, female, qol, education, premarital

## Abstract

Background

A happy and satisfied marriage is the result of two happy spouses. Getting premarital education is one of the most significant reasons for marital and sexual satisfaction. This study aimed to assess the effect of premarital education on the quality of life of Saudi women.

Methodology

A cross-sectional study was conducted on 596 Saudi women married for ≤15 years selected from the general population. Data on participants’ demographics were collected, and the quality of life was assessed using the World Health Organization Quality of Life-BREF questionnaire (WHOQOL-BREF).

Results

Only 37.2% of the participants had premarital counseling and education, even though 86.4% thought it was crucial before marriage. When this study was conducted, most participants with shorter mean marriage durations had received premarital education or counseling. The mean WHOQOL-BREF score, which measures the quality of life, was considerably higher for participants who indicated that premarital education significantly impacted the quality of their marriage and those who received premarital counseling or attended any form of premarital education.

Conclusions

Even though premarital education was viewed favorably, only 37.2% of couples obtained it. There is a need to increase public awareness of premarital education’s significance and incorporate it into the education curriculum due to the positive associations between receiving it and a higher quality of life.

## Introduction

The quality of one’s marriage or sexual relationship often determines how happy they are [1], with marriage satisfaction being a crucial factor in overall health-related quality of life (HRQoL) [2]. One of the numerous aspects that affect marriage is sexual relationship satisfaction, which is one of the most critical contributors to marital relationships [3]. Intimate life dissatisfaction is one of the significant contributors to divorce and marital problems, as well as one of the most predominant elements affecting women’s health negatively [4]. Understanding and awareness of one’s sexual life can influence a couple’s interactions and dialogues. Hence, couples will be able to modify and enhance their communication patterns and find solutions to marital problems and challenges if they are more aware of this critical aspect of their relationship [5].

An Iranian study by Aliabadian et al. revealed that couples’ sexual education positively impacted their attitudes and knowledge about sex in marriage [5]. Another Iranian study concluded that the sexual aspect of life is a crucial component of overall quality of life [6]. Further, it is critical to consider how knowledge, opinions, and beliefs from one’s personal life affect marriages and the emotional instability of partnerships. Strengthening marriages and avoiding family dissolution can be achieved by teaching couples about their health and optimal physiological functioning via sexual health education [7]. As one of the most essential educational demands, educating about sexual activity at the right time has been discussed in several Iranian studies [8-13]. We believe this has to be effectively considered because young women in our culture are highly susceptible to marriage.

According to a study by Ramezani et al., 15-50% of women are unhappy with their sexual activity. The same study also found that 50% of divorces are due to sexual life dissatisfaction being the main reason [14]. Sexual life dissatisfaction is also a widespread issue among African women, and according to studies conducted in Nigeria [15] and Ghana [16], only about half of the women (45.8% and 34%, respectively) experience joyful sexual life engagement.

Previous studies revealed that improving couples’ sexual life leads to happier marriages [17,18]. Therefore, partners must have access to counseling resources before serious marital issues occur [19]. HRQoL refers to the perceptions of well-being, optimal, and life satisfaction, which are now increasingly utilized to evaluate various treatment outcomes [20].

In Saudi Arabia, the association between premarital education and the quality of women’s life is yet to be examined. Given the differences across populations of different ethnicities, it is essential to understand whether premarital counseling is associated with happier marriages, specifically in the Saudi population. Therefore, this study aims to assess the effect of premarital education on the quality of life of women in the general Saudi population.

## Materials and methods

Study design and setting

This cross-sectional study was conducted was conducted in Saudi Arabia from May to August 2022.

Study participants and sample size

Women from the general population who had access to our questionnaire were included as study participants. We included women with a marriage duration of ≤15 years, while those with a longer marriage duration were excluded. Women with longer marriage duration were excluded from the study because premarital medical screening did not become mandatory in the Kingdom of Saudi Arabia (KSA) 15 years earlier. The sample size was calculated using the formula for calculation of sample size for cross-sectional studies, where Z = 1.96, d = 0.05, and P = Q = 0.5 due to a lack of similar studies. Thus, the total sample was 596 participants.

Data collection

Two portions of a pre-designed questionnaire using Google Forms were distributed over social media platforms. The first was to compile information on the sociodemographic characteristics of the individuals (age, nationality, marital status, educational level, and region of residence). The second section of the questionnaire included the assessment of HRQoL using the World Health Organization Quality of Life-BREF (WHOQOL-BREF) [21]. The questionnaire contains 26 questions, with two general questions and 24 items to assess the four HRQoL domains, including physical health, psychological health, social relationships, and environmental factors. The internal consistency reliability of the WHOQOL-BREF and its four domains ranged from 0.66 to 0.92, and its content validity, construct validity, and reliability were reported to be good. Higher WHOQOL-BREF scores indicate a better quality of life [20].

Ethical considerations

Ethical approval for the study was obtained from the Biomedical Research Ethics Unit of King Abdulaziz University, Jeddah, Saudi Arabia, and consent to participate in the study was obtained at the beginning of the study.

Data analysis

SPSS version 26 (IBM Corp., Armonk, NY, USA) was used for data analysis. The chi-squared test (χ^2^) was applied to qualitative data that were expressed as numbers and percentages to examine the relationship between the variables. The Mann-Whitney and Kruskal-Wallis tests were used to analyze non-parametric variables, and quantitative data were presented as mean and standard deviation (SD). Spearman’s test was used for correlation analysis, and a p-value of less than 0.05 was considered statistically significant.

## Results

Table 1 shows that 48.5% of the participants had an age range from 20 to 30 years, while 45.3% of the participants had an age range from 31 to 40 years. Of the participants, 88.6% were Saudi nationals, 95.8% were married, 68.6% had a university degree, and 70.8% were from the western KSA region. The mean marriage duration was 6.99 ± 4.14 years, while the mean family size and number of kids were 3.9 ± 2.15 and 1.69 ± 2.46, respectively.

**Table 1:** Distribution of study participants according to their demographic characteristics, marriage duration, family size, and the number of kids (N = 596). KSA: Kingdom of Saudi Arabia

Variable	Number (%)
Age (years)	
<20	8 (1.3)
20–30	289 (48.5)
31–40	270 (45.3)
41–50	24 (4)
>50	5 (0.8)
Nationality	
Non-Saudi	68 (11.4)
Saudi	528 (88.6)
Marital status	
Not married	25 (4.2)
Married	571 (95.8)
Divorced	17 (2.9)
Single	7 (1.2)
Widow	1 (0.2)
Educational level	
Less than middle school	1 (0.2)
Middle school	6 (1)
High school degree	83 (13.9)
Diploma	5 (0.8)
University degree	409 (68.6)
Postgraduate degree	92 (15.4)
KSA region	
Central region	68 (11.4)
Eastern region	41 (6.9)
Northern region	24 (4)
Southern region	41 (6.9)
Western region	422 (70.8)
Marriage duration	6.99 ± 4.14
Family size	3.9 ± 2.15
Number of kids	1.69 ± 2.46

Table 2 shows that most of the participants (86.4%) agreed that education before marriage is very important for the quality of the marital relationship other than a government premarital check. Only 37.2% underwent some premarital counseling or education other than a government premarital check. Of the participants, 16.9%, 26.7%, and 8.2% had attended premarital education workshops, courses, or conferences, respectively, while 44.6% read premarital educational books, and 53.4% visited premarital education websites/forums. Those who attended any of the above forms of premarital education were asked to rate how much they think premarital education contributed to improving the quality of their marital life, and the mean response rate from 0 to 10 was 5.14 ± 3.45.

**Table 2:** Distribution of study participants according to their responses to items related to premarital counseling or education (N = 596).

Variable	Number (%)
Importance of education before marriage on the quality of marital relationship (other than government premarital check)	
Not at all important	8 (1.3)
Not important	5 (0.80
Neutral	25 (4.2)
Important	43 (7.2)
Very important	515 (86.4)
Have you done any kind of premarital counseling or education? (Other than government premarital check)	
No	347 (62.8)
Yes	222 (37.2)
Have you attended any premarital education workshops? (Other than government premarital check)	
No	496 (83.4)
Yes	99 (16.9)
Have you attended any premarital education courses? (Other than government premarital check)	
No	437 (73.3)
Yes	159 (26.7)
Have you attended any premarital education conferences? (Other than government premarital check)	
No	547 (91.8)
Yes	49 (8.2)
Have you read any premarital educational books? (Other than government premarital check)	
No	330 (55.4)
Yes	266 (44.6)
Have you visited any premarital education websites/forums? (Other than government premarital check)	
No	278 (46.6)
Yes	318 (53.4)
If you attended any of the above or others for premarital education, how much do you think this contributed to improving the quality of your marital life? (Rate your response from 0 to 10)	5.14 ± 3.45

**Figure 1:**
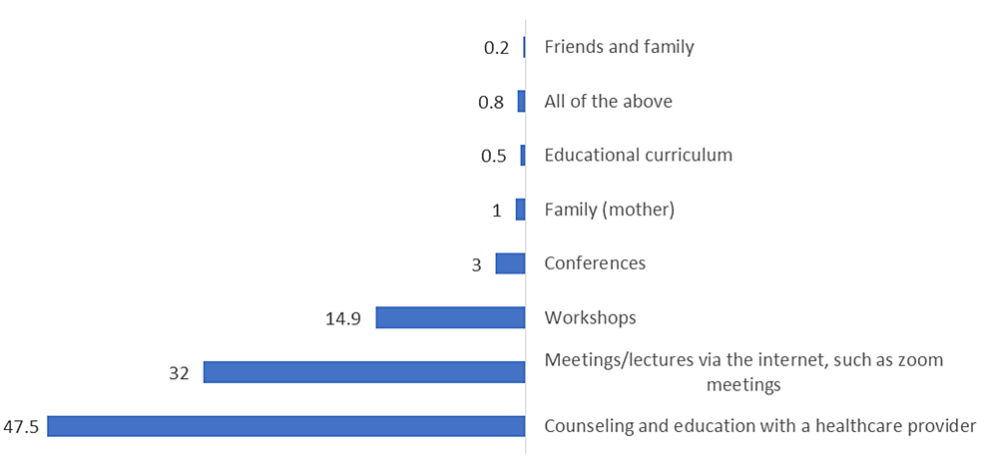
Percentage distribution of study participants according to their opinion about the best/most appropriate way of receiving premarital counseling and education.

Figure 1 demonstrates that according to the participants, the best/most appropriate ways were receiving premarital counseling and education counseling and education with a healthcare provider (47.5%) and meetings/lectures via the internet, such as zoom meetings (32%).

The mean WHOQOL-BREF score was 68.85 ± 13.75. Table 3 shows that participants with age >50 years, those of non-Saudi nationality, and those with a low middle school education had a significantly lower mean WHOQOL-BREF score, denoting a lower quality of life (p ≤ 0.05). Conversely, a non-significant relationship was found between the WHOQOL-BREF mean score and participants’ marital status or region of residency (p ≥ 0.05).

**Table 3:** Relationship between WHOQOL-BREF mean score and participants’ demographic characters (No.:596) WHOQOL-BREF = World Health Organization Quality of Life-BREF; KSA = Kingdom of Saudi Arabia

Variable	WHOQOL-BREF score	Test	P-value
Age (years)			
<20	65.87 ± 24.4	4*	0.02
20–30	70.25 ± 12.88		
31–40	67.18 ± 13.78		
41–50	73.66 ± 12.49		
>50	59.6 ± 29.84		
Nationality		3.34**	0.001
Non-Saudi	64.08 ± 12.69		
Saudi	69.46 ± 13.77		
Marital status		1.43**	0.15
Not married	66.28 ± 11.77		
Married	68.96 ± 13.83		
Divorced	63.94 ± 9.35	3*	0.228
Single	70.85 ± 16.47		
Widow	74 ± 0.001		
Married	68.96 ± 13.83		
Educational level		4*	0.003
Less than middle school	65.27 ± 14.84		
Middle school	54.83 ± 22.44		
High school degree	60.34 ± 11.01		
Diploma	79.6 ± 19.47		
University degree	68.82 ± 13.43		
Postgraduate degree	72.58 ± 11.74		
KSA region		2*	0.134
Central region	66.84 ± 14.05		
Eastern region	68.85 ± 13.28		
Northern region	67.11 ± 12.35		
Southern region	68.13 ± 10.54		
Western region	69.48 ± 13.67		

**Figure 2:**
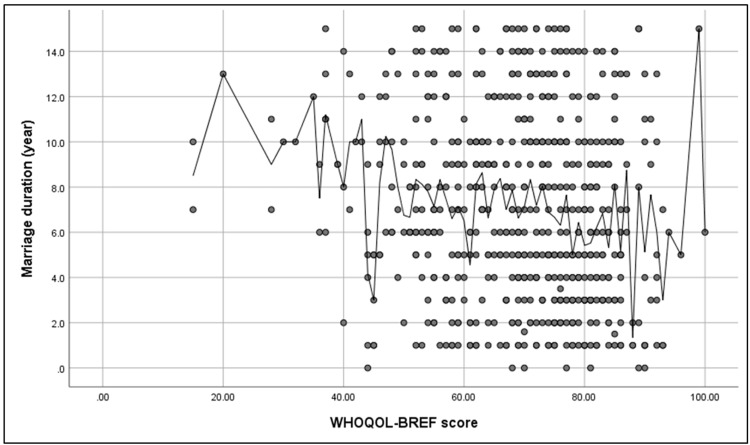
Spearman’s correlation analysis between WHOQOL-BREF score and participants’ marriage duration (N = 596). WHOQOL-BREF = World Health Organization Quality of Life-BREF

Figure 2 illustrates that a significant negative correlation was found between the WHOQOL-BREF score and participants’ marriage duration (r = -0.16, p ≤ 0.001).

As shown in Table 4, a significant negative correlation was found between the WHOQOL-BREF score and participants’ family size and the number of kids (p ≤ 0.05), while a significant positive correlation was found between the WHOQOL-BREF score and participants’ rating of how premarital education contributed to improving the quality of marital life (p ≤ 0.05).

**Table 4:** Spearman’s correlation analysis between WHOQOL-BREF score and participants’ family size, number of kids, rate of the importance of education before marriage on the quality of the marital relationship, and rate of how premarital education contributed to improving the quality of marital life (N = 596). WHOQOL-BREF = World Health Organization Quality of Life-BREF

Variable	WHOQOL-BREF score	
	R	P-value
Family size	-0.13	0.001
Number of kids	-0.16	0.001
Importance of education before marriage on the quality of the marital relationship (other than government premarital check) (rate from 1–5)	0.05	0.2
If you attended any of the above or others for premarital education, how much do you think this contributed to improving the quality of your marital life? (Rate your response from 0 to 10)	0.35	<0.001

Participants who underwent some kind of premarital counseling or education had a significantly shorter mean marriage duration compared to the other group (p ≤ 0.05), while a non-significant relationship was found between having a kind of premarital counseling or education and participants’ demographic characteristic, family size, or the number of kids (p ≥ 0.05) (Table 5).

**Table 5:** Relationship between having premarital counseling or education and participants’ demographic characteristics, marriage duration, family size, and the number of kids (N = 596). KSA = Kingdom of Saudi Arabia

Variable	Had premarital counseling or education		Test	P-value
	No, Number (%)	Yes, Number (%)		
Nationality			0.12	0.723
Non-Saudi	44 (11.8)	24 (10.8)		
Saudi	330 (88.2)	198 (89.2)		
Marital status			1.95	0.162
Not married	19 (5.1)	6 (2.7)		
Married	355 (94.9)	216 (97.3)		
Divorced	15 (4)	2 (0.9)	6.58	0.086
Married	355 (94.9)	216 (97.3)		
Single	3 (0.8)	4 (1.8)		
Widow	1 (0.3)	0 (0.0)		
Educational level			5.74	0.332
Less than middle school	1 (0.30	0 (0.0)		
Middle school	5 (1.3)	1 (0.5)		
High school degree	59 (15.8)	24 (10.8)		
Diploma	2 (0.5)	3 (1.4)		
University degree	250 (66.8)	159 (71.6)		
Postgraduate degree	57 (15.2)	35 (15.8)		
KSA region			2.33	0.674
Central region	40 (10.7)	28 (12.6)		
Eastern region	26 (7)	15 6.8)		
Northern region	16 (4.3)	8 (3.6)		
Southern region	22 (5.9)	19 (8.6)		
Western region	270 (64)	152 (68.5)		
Marriage duration	7.69 ± 4.16	5.81 ± 3.82	5.36*	<0.001
Family size	3.9 ± 1.83	3.9 ± 2.6	0.87*	0.379
Number of kids	1.79 ± 2.91	1.53 ± 1.4	1.18*	0.236

**Figure 3:**
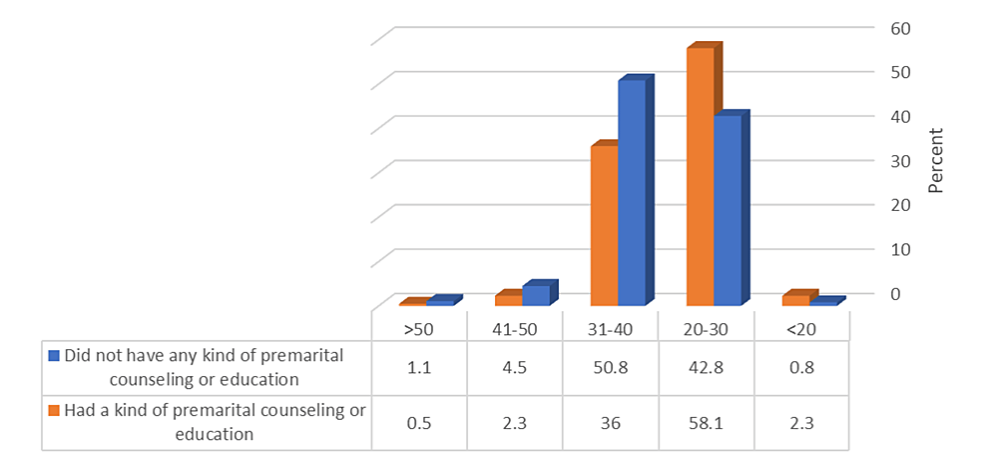
Relationship between having premarital counseling or education and participants’ age (N = 596).

Figure 3 demonstrates that younger participants (20-30 years) had a significantly shorter mean marriage duration compared to that of the other group (p ≤ 0.05).

Table 6 shows that participants who rated education before marriage on the quality of the marital relationship other than government premarital check as very important had a significantly higher mean WHOQOL-BREF score, indicating a better quality of life (p ≤ 0.05). At the same time, participants who had some kind of premarital counseling or education and who attended any premarital education workshops, courses, or conferences had a significantly higher mean WHOQOL-BREF score, indicating a better quality of life (p ≤ 0.05).

**Table 6:** Relationship between WHOQOL-BREF mean score and participants’ response to items related to premarital counseling or education (No.:596) WHOQOL-BREF = World Health Organization Quality of Life-BREF

Variable	WHOQOL-BREF score	Test	P-value
Importance of education before marriage on the quality of the marital relationship (Other than government premarital check)		4*	0.023
Not at all important	48.75 ± 25.44		
Not important	56.8 ± 11.6		
Neutral	67.76 ± 12.85		
Important	71.37 ± 8.67		
Very important	69.12 ± 13.67		
Have you done any kind of premarital counseling or education? (Other than government premarital check)		6.65**	<0.001
No	66.27 ± 13.03		
Yes	73.2 ± 13.88		
Have you attended any premarital education workshops? (Other than government premarital check)		2.68**	<0.001
No	68.02 ± 13.34		
Yes	73.01 ± 15.04		
Have you attended any premarital education courses? (Other than government premarital check)		5.01**	<0.001
No	67.37 ± 13.19		
Yes	72.93 ± 14.46		
Have you attended any premarital education conferences? (Other than government premarital check)		1.78**	0.074
No	68.61 ± 13.28		
Yes	71.59 ± 18.16		
Have you read any premarital educational books? (Other than government premarital check)		1.08**	0.28
No	68.33 ± 13.85		
Yes	69.5 ± 13.63		
Have you visited any premarital education websites/forums? (Other than government premarital check)		0.33**	0.735
No	68.65 ± 14.2		
Yes	69.1 ± 13.37		

## Discussion

This study aimed to evaluate the degree to which premarital education is associated with the quality of life of female partners. According to our findings, slightly less than half of the participants were between the ages of 20 and 30, which compared favorably with that reported in a Bahraini study by AlRoomi et al. [22]. The average participant had a university degree, and 70.8% of the participants were from the western KSA region. The mean marriage duration in this study was 6.99 ± 4.14 years. In a study from Minnesota, 48% of the participants reported their marriage as being more than 10 years in duration [23]. According to this study, the average family size was 3.9 people with 2.15 children, with 1.69 and 2.46 children overall. This can be explained by the fact that many small families and newlywed couples are interested in education and research that can significantly alter their life.

In the most recent survey, 37.2% of the participants underwent premarital education or counseling that was not provided by the government. Overall, 53.4% of respondents cited premarital education websites or forums as their primary source of information, while 44.6% cited premarital education books as their primary source of information. Furthermore, premarital education conferences, workshops, or classes were attended by 16.9%, 26.7%, and 8.2% of the population, respectively. This outcome was consistent with findings from the research done in Iran by Moodi et al. [24]. The survey found that books accounted for 48.7% of the primary information sources, followed by parents (20.6%) and other sources [24]. Another study conducted in Egypt by Ali et al. revealed that social media ranked second (58.1%) after school/faculty as the primary information source [25]. These variations may be due to the difference in the surrounding environment, customs, and traditions as well as the fear of asking about marriage by any unengaged individuals. Further, this study demonstrated that according to the participants, the most appropriate way of receiving premarital counseling and education was getting it from a healthcare provider (47.5%).

According to a prior study, participants who were married before scored better on knowledge tests than those who had never been married [26]. The WHOQOL-BREF mean score and individuals’ marital status did not, however, show a significant correlation in this study, which may be because of their prior training in screening programs, or their prior marital experience.

The WHOQOL-BREF mean score and participants’ regions of residence did not significantly correlate. This result is consistent with that from investigations carried out in Iran by Mahmoodi and Egypt by Farahat et al. [27,28], where there was no statistically significant difference between rural and urban locations regarding premarital care awareness rates. The WHOQOL-BREF score and premarital education showed a strong favorable connection in this study, with other studies supporting our findings. The mean score of the couples in knowledge, attitude, and marital happiness increased following the educational intervention, according to studies conducted in Iran [24,29]. According to a different study, couples counseling made a considerable improvement in both genders [28]. In the United States, a prior study found that participants who underwent premarital counseling reported better levels of marital life satisfaction than those who did not receive any counseling [23].

Participants in this study who assessed education before marriage on the quality of the marital relationship as extremely essential in addition to the government premarital check had a significantly higher mean WHOQOL-BREF score. Participants who received premarital education or counseling also had a significantly higher mean WHOQOL-BREF score, indicating a higher quality of life. These findings are in line with those from the research conducted in Iran by Parhizgar et al. and Moodi et al. [24,29]. According to these studies, premarital counseling and marriage satisfaction are significantly correlated. The knowledge and attitudes of those who were monitored in the areas of reproductive health and family planning before and after participating in premarital counseling or education also showed a substantial difference. Furthermore, premarital education has been shown to improve quality of life, underscoring the necessity of including this subject in high school and university curricula [24,29].

The limitation of this study is that the pre-designed questionnaire could have a recall bias.

## Conclusions

This study concluded that most participants believed that education before marriage is crucial. However, only 37.2% had some kind of premarital counseling or education other than a government premarital check. The WHOQOL-BREF score and participants’ assessments of how premarital education improved the quality of their marriages showed a strong positive association. No study examined this association in the Saudi Arabian population; therefore, these findings lay an essential basis for future studies on similar people from other centers across the KSA. Such studies can help determine the effect of premarital education on both men and women. Moreover, it is necessary to raise awareness among the general population about the value of premarital counseling through official and informal education and media coverage.

## Figures and Tables

**Figure 4:**
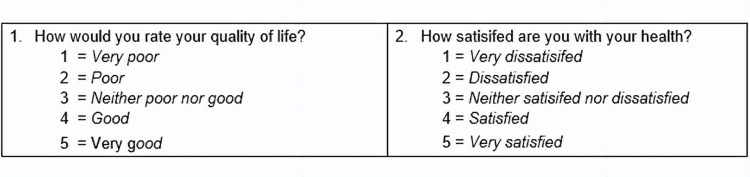
The first two general questions.

**Figure 5:**
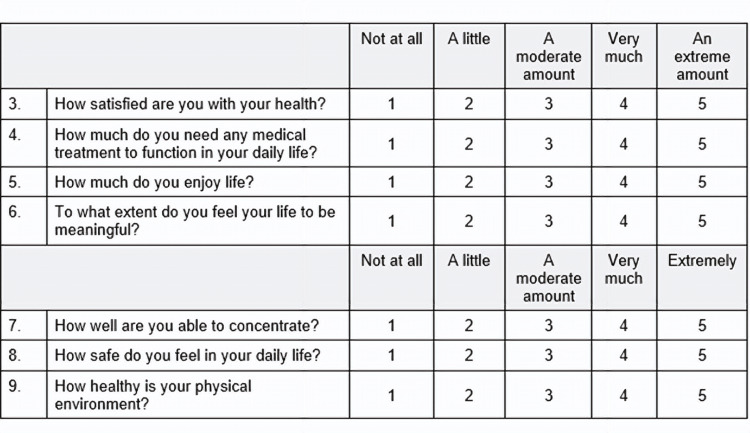
Questions that inquire about the extent of experiencing particular items.

**Figure 6:**
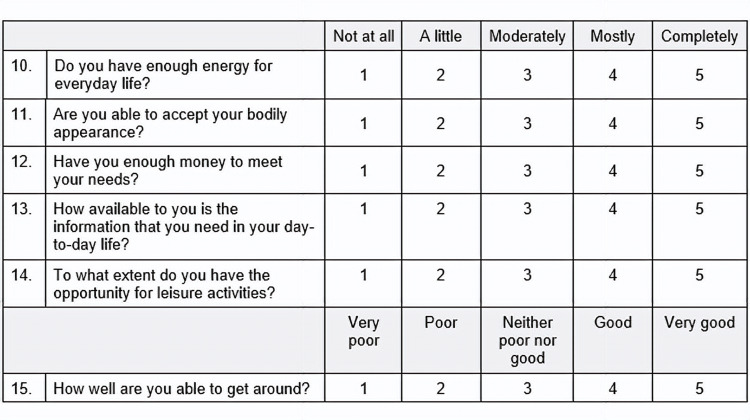
Questions about how completely you have been through some things.

**Figure 7:**
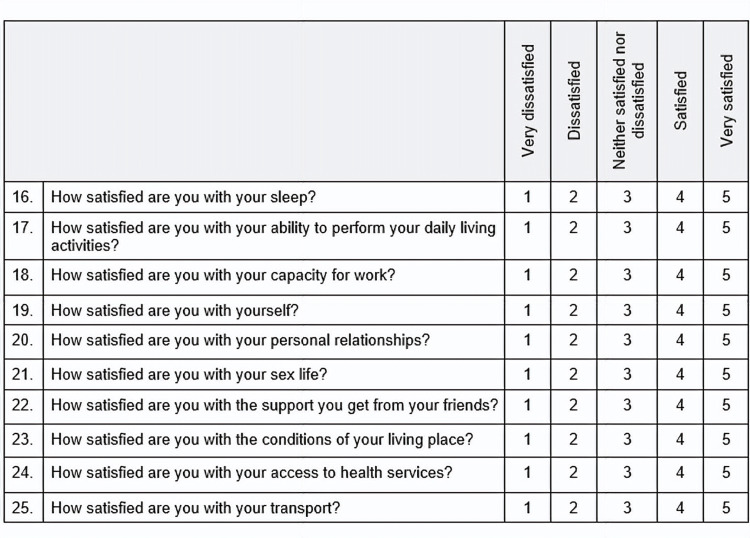
Questions ask how satisfied you have felt over the previous two weeks regarding various parts of your life.

**Figure 8:**
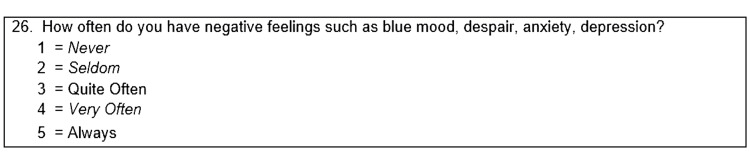
The last question asks how often you have felt or experienced certain things in the last two weeks.

## Appendices

The World Health Organization Quality of Life-BREF (WHOQOL-BREF) questionnaire is shown in the following figures.
