# Correction: Effect of Premarital Education on the Quality of Life of Female Partners: A Cross-Sectional Study

**DOI:** 10.7759/cureus.c84

**Published:** 2022-12-14

**Authors:** Nedaa M Bahkali, Ghaida A Eissa, Farah M Alharbi, Fatmah A Alzahrani, Fawaz E Edris, Nahla K Ibrahim

**Affiliations:** 1 Obstetrics and Gynecology, King Abdulaziz University, Jeddah, SAU; 2 Medical School, King Abdulaziz University, Faculty of Medicine, Jeddah, SAU; 3 Medical School, King Abdulaziz University Hospital, Jeddah, SAU; 4 Obstetrics and Gynecology, Umm Al Qura University, Makkah, SAU; 5 Epidemiology, King Abdulaziz University, Jeddah, SAU

This article has been corrected to remove a duplicate paragraph in the Discussion and change the following incorrect citation from 3 to 24: "Furthermore, premarital education conferences, workshops, or classes were attended by 16.9%, 26.7%, and 8.2% of the population, respectively. This outcome was consistent with findings from the research done in Iran by Moodi et al. [3]." This now reads as follows: "Furthermore, premarital education conferences, workshops, or classes were attended by 16.9%, 26.7%, and 8.2% of the population, respectively. This outcome was consistent with findings from the research done in Iran by Moodi et al. [24]."

